# Norm values and psychometric properties of the short version of the Trier Inventory for Chronic Stress (TICS) in a representative German sample

**DOI:** 10.1371/journal.pone.0222277

**Published:** 2019-11-18

**Authors:** Katja Petrowski, Sören Kliem, Cornelia Albani, Andreas Hinz, Elmar Brähler

**Affiliations:** 1 Clinic of Psychotherapy and Psychosomatic Medicine, Technische Universität Dresden, Dresden, Germany; 2 Medical Psychology and Medical Sociology; Clinic of Psychosomatic Medicine and Psychotherapy, University Medical Center of the Johannes Gutenberg University of Mainz, Mainz, Germany; 3 Criminological Research Institute of Lower Saxony, Hannover, Germany; 4 Department of Medical Psychology and Medical Sociology, University of Leipzig, Leipzig, Germany; 5 Clinic of Psychosomatic Medicine and Psychotherapy, University Medical Center of the Johannes Gutenberg University of Mainz, Mainz, Germany; University of Lleida, SPAIN

## Abstract

The Trier Inventory for Chronic Stress (TICS), consisting of 57 items, is an instrument for measuring chronic stress in nine areas. There is also a short form (SSCS) of the TICS consisting of 12 items. However, this 12-item short form does not include all nine areas of the theoretical model and the long version. Therefore, a short version including all nine scales/areas was investigated. The TICS was taken by a sample of *N* = 2,473 respondents from the general population, aged 14 to 99, selected by random-route sampling. Confirmatory factor analyses applying robust maximum likelihood estimations (MLM) tested the model fit. The one-factor-model proposed by the original authors was tested, and the SSCS showed an unacceptable model fit. For the development of an economical short version of the TICS, including items of the nine areas of chronic stress, nine items based on the alphamax algorithm were selected. The one-factor-model of this new short version of the TICS of nine items provided a good fit for the latent construct and showed good reliability (α = .88). A new and reliable short version of the TICS consisting of only 9 items representing the 9 scales/areas for the assessment of chronic stress was identified to possess a good model fit and good reliability.

## Introduction

According to the stress report in 2012, the level of perceived stress and health complaints has increased since the last data collection in 2005/2006 [1]. The consequences of long-lasting or chronic stress lead to an increased risk for impaired psychological wellbeing and acute physical illnesses [2, 3]. Hereby, prospective observational studies provide support for stress as an important factor in certain diseases but cannot establish a causal relationship [4]. Clear associations between psychological stress and disease have been established for depression, cardiovascular disease, sleep disorder, chronic pain, and HIV/AIDS [4, 5, 6, 7, 8, 9]. Other areas in which evidence for the role of stress is beginning to emerge include upper respiratory tract infections, asthma, herpes viral infections, autoimmune diseases, and wound-healing [9]. Thus, chronic stress and non-acute stress is associated with clinically relevant health impairments based on stress research findings. Therefore, measuring chronic stress with a short instrument is of relevance to a wide research field [10].

Cohen and colleagues [3] provided a thorough overview of assessment instruments concerning general stress instruments such as the Perceived Stress Scale [11], the Stress Reactivity Scale [12], and the Perceived Stress Questionnaire [13, 14]. They also summed up area-specific instruments to assess stress at the workplace such as Occupational Stress Indicator [15] and the Job Content Questionnaire [16], or in the marital or family domain, e.g., the Family Environment Scale [17]. With these instruments, acute stress in general or in specific areas can be assessed.

When the organism no longer manages to meet the increasing demands and pressures of acute stress, stress can become chronic and has a larger impact on the health than acute stress [18]. To measure the general chronic stress, the Perceived Stress Questionnaire [13, 14] is well established with different time references to four, six, and eight weeks, and one year. The Trier Inventory for Chronic Stress (TICS) by Schulz, Schlotz, and Becker [19] is the first instrument that explicitly captures area-specific chronic stress all in one questionnaire including Work Overload, Social Overload, Pressure to Perform, Work Discontent, Excessive Demands from Work, Lack of Social Recognition, Social Tensions, Social Isolation, and Chronic Worrying. The basis for the questionnaire is the Systemic Requirement Resource Model of Health [20], which proposes that an individual’s health may be promoted and preserved when the individual can depend on the availability of internal and external resources to satisfactorily deal with internal and external demands [20]. In accordance with the systemic-requirement-resource model of health [20], these nine factors are measured by 57 items which can also be grouped into the two higher order factors High Demands and Lack of Satisfaction. The High Demands captures the specific working conditions and the Lack of Satisfaction refers to an individual’s need as well as to unsatisfactory working conditions and social conditions [19, 21]. As the TICS scales were developed based on the systemic-requirement-resource model of health [18], the authors postulated content validity as a logical consequence [19]. In addition, the associations with depressive symptoms (Beck Depression Inventory; [22]) was investigated to test discriminant validity since chronic stress is strongly related to depressive symptoms [23]. All of the TICS scales are highly correlated with the depression scale (.26 - .63), except for the scale Pressure to Perform (.11). Only the scale Social Overload but not Work Overload correlated with negative sleep quality and appetite which are other symptoms of the depression [24, 25]. The results of a confirmatory factor analysis (CFA) in a representative German study (based on gender, age, income and location of living) showed evidence for a good factorial validity of the theoretical proposed nine and two factorial model [21].

Due to the necessity for a brief inventory for the assessment of chronic stress in practice, the 12-item short screening scale of the Trier Inventory of Chronic Stress (Short Screening Scale for Chronic Stress, SSCS) was developed [19]. Therefore, a representative sample (N = 604) of the German population (based on the location of living) was drawn. In the selected locations of Germany, then, people were randomly approached by letter based on the telephone book. Since the two-factor model showed a strong, unrotated first factor, the items with the highest loadings on this strong first factor were chosen for the 12-item short screening scale. However, these items were only originated from five of the nine stress areas: Chronic Worrying, Work Overload, Social Overload, Excessive Demands of Work, and Lack of Social Recognition. The internal consistency of the 12-item SSCS was reported with a Cronbach’s alpha = .91 [19, 21]. For the construct validity of the 12-item SSCS, the original authors used different general stress survey measures, and these correlations (between r = .49 and r = .75) provided evidence for the convergent validity of the SSCS [19]. In reference to the 57-item long version of the TICS, the five scales Chronic Worrying, Work Overload, Excessive Demands from Work, Lack of Social Recognition, and Social Overload, whose items were included, correlated higher with the 12-item SSCS (*r* = .68-.87), except for Social Overload with a lower correlation (*r* = .45). The four scales Pressure to Perform, Work Discontent, Social Tensions, and Social Isolation, whose items were not included, correlated lower with the 12-item SSCS (*r* = .40 - .56) [19].

In the 12-item SSCS only five of the nine theoretically proposed areas of chronic stress are underrepresented with this short chronic stress screening instrument. This procedure of item selection (highest loading) has inadvertently narrowed the content domain and has lessened the validity of the short screening form [26]. Thus, the strength and exceptionality of the area-specific chronic stress assessment of the TICS based on the theoretical model is not represented in the short screening scale anymore. Therefore, the aim of the present study was to identify a short version of the TICS, which represents the theoretically and empirically driven nine areas of chronic stress. Therefore, a different empirical approach should be used such as the alphamax macro [27,28]. This is a scale-shortening algorithm that establishes combinations of items that would result in a good coefficient alpha. By this approach a short form of the TICS can be identified that captures all nine areas of chronic stress so that all areas can be assessed economically.

## Methods

### Sample

The data collection was conducted by the ‘USUMA’ polling institute Berlin, Germany on behalf of Leipzig University. The households and the participants asked to fill out several questionnaires were selected by the random-route sampling method. The random-route sampling method is a national method based on a combination of random, systematic, and stratified probability samples at different levels. First, a stratified selection of sampling units is carried out from which a systematic selection of households takes place during random walks. In the end, one person per household is randomly selected via Kish-table [29]. The representativeness of the sample is then validated based on information obtained from the German Federal Statistical Office [30]. The response rate of the present sample was 62.3%, resulting in a basic sample of *N* = 2,607 participants. The exclusion of non-native speakers and incomplete data sets resulted in an acceptable loss of *N* = 134 cases (5.4% of all cases). The final sample consisted of *N* = 2,473 cases at a mean age of *M* = 47.9 (*SD* = 17.9, range = 14–99), including 52.6% female participants. Other socio-demographic details concerning the basic sample, including those participants who had not completed the questionnaire, that were already reported elsewhere [21]. Written informed consent was obtained from all the participants. Each participant received a data protection declaration, which is in agreement with the Helsinki Declaration. The study followed the ethical guidelines of the “German Professional Institutions for Social Research” [30] as well as by the ethics committee of the University of Leipzig, Germany. Ethics reference number is 050-13-11032013.

### Instruments

The *Trier Inventory for Chronic Stress* (TICS) [19] is a standardized questionnaire for assessing nine interrelated factors of chronic stress. The participants rate the 57 items on a five-point rating scale in respect to how often they encountered a certain situation or had a certain experience within the previous three months (0 = never, 1 = rarely, 2 = sometimes, 3 = often, 4 = very often). Internal consistency (Cronbach’s Alpha), as reported for the original samples by Schulz and colleagues (2004) [19] with a mean of α = .87 and a range from .84 to .91, indicates a good to very good reliability.

### Development of the nine-item short form of the TICS

The criteria for the short scale were a very good coefficient alpha value, the uni-dimensionality of the instrument (which is necessary for calculating a total score), and a small number of items to provide an economic measurement. Furthermore, to retain the construct validity of the short form, it was preferable for the new scale to contain one item each of the originally postulated dimensions. The alphamax macro [27, 28], a scale-shortening algorithm, was used to establish combinations of items that would result in a good coefficient alpha. The algorithm computes Cronbach’s alpha for every possible subset of a given number of items (nine in the present study). These item sets with a high coefficient alpha were then evaluated further.

A confirmatory factor analysis (CFA) was conducted and the item set tested. The CFA estimation followed the procedure mentioned in the statistical procedure. Based on these analyses, a short scale was developed that included nine items (one item of each of the originally postulated factors).

### Statistical procedure

The internal consistency of the TICS-9 and TICS-12 is reported as Cronbach’s α-coefficient. The selectivity as the correlation of the item with the sum of all other items is determined: Item difficulty coefficients are calculated as quotients of the sum of the item values that were obtained and the sum of the maximum achievable item values multiplied by 100.

A confirmatory factor analysis (CFA) was conducted to test for the one-factor solution of the TICS-9 and TICS-12. Given the violation of multivariate normality assumption, we used the Satorra and Bentler’s [31] scaling method (MLM). To evaluate the goodness of fit of the relevant model, we considered three different criteria: a) the robust version of the root mean square error of approximation (robust RMSEA) as well as the 90% confidence interval assess absolute model fit, the two additional calculated criteria (robust version of the Comparative Fit-Index [robust CFI] and the robust version of the Tucker Lewis Index [robust TLI]) are measurements of relative model-fit, compared to the “null” model. RMSEA values < .050 represent a “close fit”, RMSEA values between .050 and .080 represent a “reasonably close fit”, and RMSEA values > .100 represent an “unacceptable model” [32]. Regarding CFI and TLI, Hu and Bentler [33] suggested a CFI and TLI > .950 for a good model fit. To further test the reliability of the factor models Mc Donald’s coefficient omega (ω) was computed for each of the subscales as well as the overall sum score.

**Fig 1:**
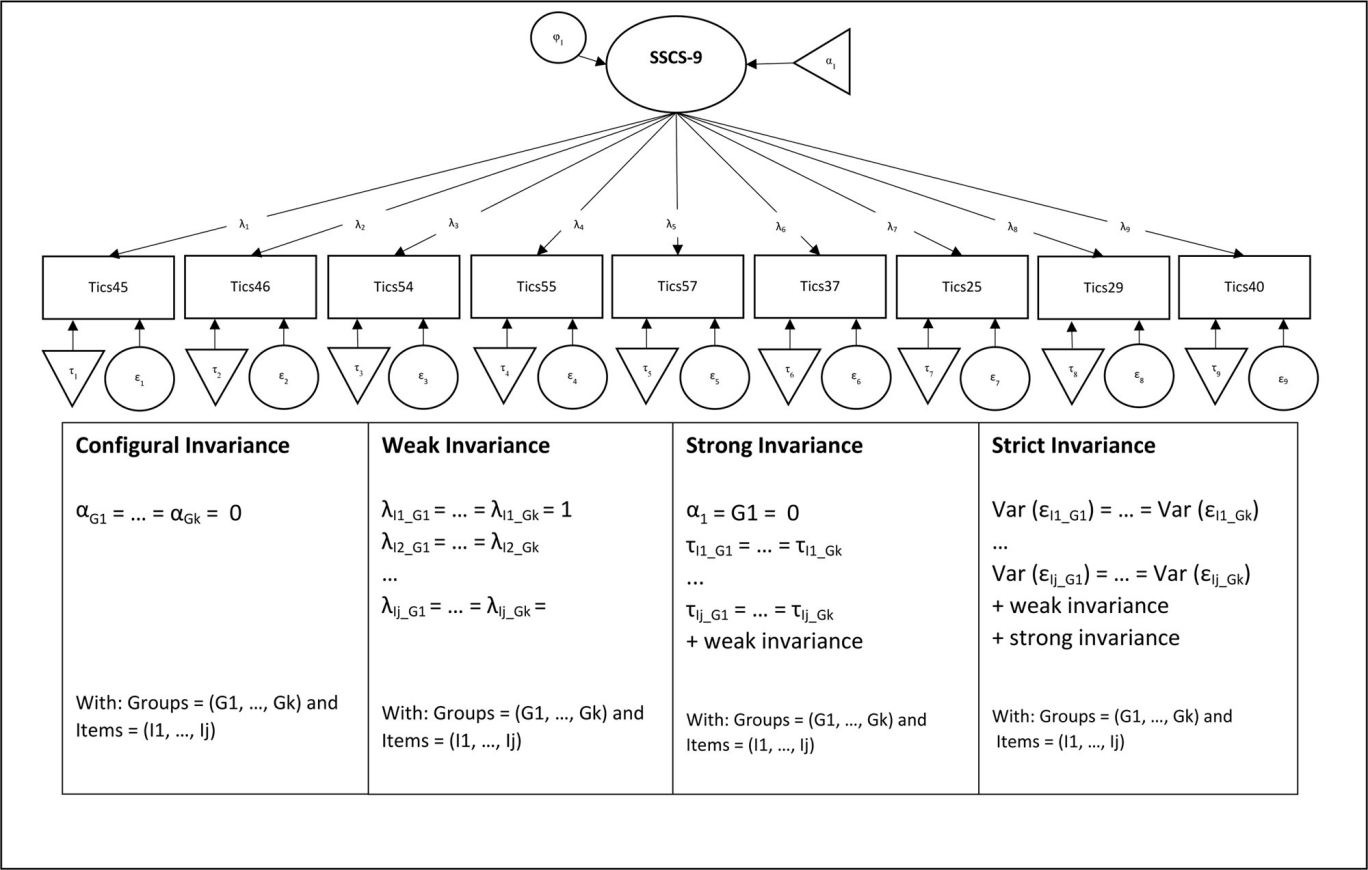
Models relevant for the invariance test.

Furthermore, measurement invariance tests using multi-group factor analyses were conducted across gender (group 1 = men; group 2 = women), and age (group 1: < 25 years of age; group 2: 25 to 34 years of age; group 3: 35 to 44 years of age; group 4: 45 to 54 years of age; group 5: 55 to 64 years of age; group 6: 65 to 74 years of age; group 7: ≥ 75 years of age). Measurement invariance tests were performed using the sequential strategy discussed by Meredith and Teresi [34]: First, we tested a configural invariance model, e.g., which item load on which factor is imposed on the subgroups. Configural invariance refers to the equivalence of the factorial structure. It is given if the analyzed constructs show the same dimensionality and, in addition, the observed variables are correlated with the same latent constructs in both groups. Configural invariance is necessary but not sufficient for expecting an unbiased comparison of measurements between groups. Second, we tested the weak invariance model by constraining the estimate factor loadings to be equal across groups. If empirical support for weak invariance is provided, it allows the comparison of structural relationships (e.g., correlation coefficients, structural [path] coefficients) between latent constructs in groups. Third, the strong invariance model was tested by constraining both intercepts and loadings to be equal across groups. This level of invariance allows the comparison of means of the latent construct between groups. Finally, we tested the strict invariance model by constraining the loading, intercepts, and item error variances to be equal across groups. Different residual variances in groups can have two possible consequences. First, it can lead to different reliabilities of indices in those groups. Second, it can affect decisions in screening processes that depend on the expression of a construct, resulting in different error rates (e.g. sensitivity, specificity) for different groups (e.g., [35], please see Fig 1 for further details). As noted by Chen [36], the commonly used chi-square differences tests of nested models is almost always significant in large samples and highly sensitive to departures from multivariate normality. Thus, we used robust CFI differences (ΔCFI) as well as robust RMSEA differences (ΔRMSEA) to compare the difference stages of measurement invariance. As recommended by Chen [37], a change of > .010 in ΔCFI, supplemented by a change of ΔRMSEA < -0.015, was regarded as indicative of non-invariance. The data analysis was carried out in R using the packages lavaan and semTools [38, 39].

## Results

### Descriptive item analysis of the 12-Item SSCS

For item characteristics such as skewness and kurtosis, see Table 1. The 12 items selected for the SSCS show an item-rest correlation between .55 and .70. For the SSCS, the internal consistency was good to very good with Cronbach’s Alpha = .91 (*N* = 2,411). A complete overview of the mean values and standard deviations as well as the inter-correlations of the complete TICS items can be found in the supplementary S1 Table.

**Table 1:** The 12-item SSCS and the new nine-item short version of the TICS and its item discriminative power (N = 2,473).

*SSCS*	*Scale*	*M*	*SD*	*Item-rest correlation*	*Skewness*	*Kurtosis*	λ
	**12-item SSCS**						
tics25	Chronic Worrying	1.10	1.03	.674	.74	-.02	.705
tics36	Chronic Worrying	1.17	1.07	.675	.64	-.34	.703
tics54	Work Overload	1.07	.95	.702	.56	-.37	.746
tics16	Chronic Worrying	1.29	1.04	.607	.45	-.50	.631
tics47	Excessive Demands from Work	1.04	.87	.611	.57	-.07	.649
tics44	Work Overload	1.18	1.02	.636	.49	-.50	.677
tics09	Chronic Worrying	1.23	.97	.549	.48	-.26	.572
tics35	Excessive Demands from Work	.95	.96	.659	.85	.21	.695
tics57	Social Overload	.96	.92	.655	.64	-.28	.696
tics18	Lack of Social Recognition	1.05	.98	.622	.66	-.22	.654
tics31	Lack of Social Recognition	1.05	.99	.618	.69	.71	.654
tics38	Work Overload	1.24	1.03	.606	.40	-.19	.648
Total		13.33	8.32	-	.33	-.47	
	**Nine-item short version of the TICS**						
tics45	Social Tensions	.87	.94	.677	.89	.13	.717
tics46	Lack of Social Recognition	1.12	1.06	.634	.63	-.40	.678
tics54	Work Overload	1.07	.95	.694	.56	-.37	.753
tics55	Excessive Demands from Work	.89	.91	.658	.85	.21	.714
tics57	Social Overload	.97	.92	.678	.64	-.28	.734
tics37	Work Discontent	1.06	.98	.643	.61	-.37	.690
tics25	Chronic Worrying	1.10	1.03	.592	.74	-.02	.632
tics29	Social Isolation	1.22	.98	.582	.49	-.31	.615
tics40	Pressure to Perform	1.13	1.01	.545	.59	-.36	.576
total		9.42	6.33	-	-.43	.10	

### Confirmatory Factor Analysis (CFA) and measurement invariance analysis of the 12-Item SSCS

Item assignments were adopted from Schulz, Schlotz, and Becker [19] and can be seen in the first column of Table 1. The proposed one-factor-model was tested. The robust version of the CFI showed a non-acceptable fit (robust CFI = .879 < .900); the robust version of the TLI fit was also not sufficient (robust TLI = .852 < .900). Finally, the robust version of the RMSEA did not fulfill the criteria for a good model fit (robust RMSEA = .111 [95%-CI: .105, .117] > .08). Overall, the 12 items as a one-factor model showed an unacceptable model fit. Due to the poor model fit, no further measurement variance analysis was performed. Composite reliability in the form of McDonald’s coefficient omega was ω  =  0.91 (for the general factor).

### Descriptive item analysis of the new nine-item short version of the TICS

In Table 1, the nine items with their item characteristics are presented. The selected items for the new TICS-short scale show item rest correlations between .54 and .69. For item characteristics such as skewness and kurtosis, see Table 1. Cronbach’s Alpha was .88. The new TICS short form highly correlates with the SSCS (*r* = .91). The correlation coefficients between the new nine-item short version of the TICS and the scales of the 57-item long form of the TICS range between r = .65 and r = .83 (Tables 2 and 3). These high correlations are higher than the correlations of the 12-item short screening scale. Therefore, these results show that the new nine-item short version of the TICS constitutes a very good representation of the general chronic stress load.

**Table 2:** The 12-item SSCS and the new nine-item short version of the TICS and their properties for different age cohorts and for gender.

***M (SD)***					
	**12-item SSCS**				
**Total sample**	**14–44 years**	**45–64 years**	**65–99 years**	**Women**	**Men**
*N = 2*,*411*	*N* = 1,112	*N* = 772	*N* = 527	*N* = 1,265	*N* = 1,146
**13.33 (8.32)**	**14.40 (8.30)**	**13.25 (8.08)**	**11.08 (8.34)**	**13.69 (8.43)**	**12.92 (8.17)**
	**nine-item short version of the TICS**				
**Total sample**	**14–44 years**	**45–64 years**	**65–99 years**	**Women**	**Men**
*N* = *2*,*422)*	*N* = 1,120	*N* = 770	*N* = 532	*N* = 1,272	*N* = 1,150
**9.42 (6.33)**	**10.35 (6.30)**	**9.47 (6.10)**	**7.29 (6.29)**	**9.56 (6.38)**	**9.29 (6.29)**

**Table 3:** Correlation coefficients (Pearson) of the new nine-item short version of the TICS, the 12-item short screening scale of the TICS and the scales of the 57-item long version of the TICS (N = 2,473).

	*New nine-item short version*	*12-item SSCS*
Work Overload	.78***	.79***
Social Overload	.70***	.68***
Pressure to Perform	.68***	.64***
Work Discontent	.78***	.74***
Excessive Demands from Work	.83***	.86***
Lack of Social Recognition	.80***	.80***
Social Tensions	.80***	.76***
Social Isolation	.65***	.63***
Chronic Worrying	.74***	.86***
Chronic Stress Screening Scale (SSCS)	.91***	

*** *p* < .001.

### Confirmatory Factor Analysis (CFA) of the new nine-item short version of the TICS

The identified items were tested for the proposed one-factor model. The robust version of the RMSEA (robust RMSEA = .058 < .080) is below the aforementioned cut-offs for reasonably close fit. In line with this, the robust version of the CFI (robust CFI = .975 > .950) and the robust version of the TLI (robust TLI = .966> .950) also show a good model fit. In addition, all standardized path coefficients (i.e., factor loadings) show values larger than λ = 0.3 (see Table 1) and are highly significant (p < .001). Therefore, the items show a good measurement for the latent construct [39]. Composite reliability in the form of McDonald’s coefficient omega was ω  =  0.88 (for the general factor).

The results of the measurement invariance analysis regarding age and gender are depicted in Table 4. Regarding gender, the baseline model (Model 0; configural invariance), which simultaneously estimated all model parameters freed across groups resulted in an excellent model fit (robust CFI = .976; robust RMSEA = 0.057). Weak invariance was examined by comparing Model 0 with Model 1 (see Table 4), which constrained all factor loadings to be invariant across aforementioned groups. ΔCFI were below the cut-off recommended by Chen. Furthermore, the model fit was excellent to good (robust CFI = 0.976; robust RMSEA = 0.053). Strong invariance was examined by comparing Model 1 with Model 2 (see Table 4), which constrained all item intercepts to be invariant across groups. Again, ΔCFI were below the cut-off recommended by Chen; the general model fit was excellent to good (robust CFI = 0.9971; robust RMSEA = 0.055). Therefore, strong invariance can be assumed. Strict invariance was examined by comparing Model 2 with Model 3, which constrained all item residual variances to be invariant across groups. ΔCFI were below the cut-off recommended by Chen. Furthermore, the model fit was excellent to good (robust CFI = 0.971; robust RMSEA = 0.051). Thus, strict invariance can be assumed for gender.

**Table 4:** Analysis of factorial invariance for age and gender using multigroup confirmatory factor analyses.

		*Χ*^*2*^_*scaled*_	*df*	CFI	ΔCFI	RMSEA	ΔRMSEA	Measurement Invariance Test^a^
**Gender** (group 1 = men; group 2 = women)								
Model 0	configural invariance	190.6	54	.976		.057		√
Model 1	weak invariance	203.8	62	.976	+.000	.053	+.004	√
Model 2	strong invariance	249.0	70	.971	+.005	.055	-.002	√
Model 3	strict invariance	252.3	79	.971	+.000	.051	+.004	√
**Age** (group 1: < 25 y; group 2: 25 to 34 y; group 3: 35 to 44 y; group 4: 45 to 54 y; group 5: 55 to 64 y; group 6: 65 to 74 y; group 7: ≥ 75 y)								
Model 0	configural invariance	347.7	189	.970		.063		√
Model 1	weak invariance	412.6	237	.969	+.001	.057	-.006	√
Model 2	strong invariance	588.8	285	.951	+.018	.066	-.009	√
Model 3	strict invariance	741.2	339	.935	+.016	.069	-.004	√

*Notes*: *df* = degrees of freedom; CFI = robust version of the Comparative Fit Index; ΔCFI = differences between models (0 and 1, 1 and 2; 2 and 3) regarding robust CFI; RMSEA = robust version of the root mean square of approximation; ΔRMSEA = differences between models (0 and 1, 1 and 2; 2 and 3) regarding robust RMSEA

^**a**^ **=** *ΔCFI* ≤ .*010* supplemented by *ΔRMSEA ≥ -*.*015* indicates non-invariance. √ marks invariance

Regarding the different age groups, the baseline model (Model 0) resulted in an excellent to good model fit (robust CFI = .970; robust RMSEA = 0.063). Weak invariance was examined by comparing Model 0 with Model 1; ΔCFI were below the cut-off recommended by Chen. Strong invariance was examined by comparing Model 1 with Model 2, resulting in a considerable worsening of model fit regarding ΔCFI = 0.018. However, the deterioration of the model fit (on the basis of the ΔCFI) was not supplemented by the relevant change in RMSEA (ΔRMSEA = -.009) so that a strong invariance may be assumed. Strict invariance was examined by comparing Model 2 with Model 3, again resulting in a considerable worsening of the model fit in CFI (ΔCFI = 0.016). However, this deterioration of the model fit (on the basis of the ΔCFI) was not supplemented again by the relevant change in RMSEA (ΔRMSEA = -.004), so that a strict invariance may be assumed.

## Discussion

The aim of the present study was to identify a short version of the TICS, which represents the theoretically and empirically driven nine areas of chronic stress based on a representative data set. In addition, the guidelines for developing a short form were followed [26].

The reliability of the 12-item short screening scale of the TICS is very high. However, the confirmation of the one-factorial structure could not be shown in this sample. Sample composition and/or unequal representation of TICS subscales (e.g. high representation of chronic worrying) might be responsible for this.

Therefore, the aim was to identify a nine-item short version for the TICS with one item from each area of the theoretical model. The alphamax algorithm [29] was used to compile a subset of items (one from each subscale) that maximizes internal reliability as quantified by Cronbach’s alpha. This nine-item short version of the TICS showed a good reliability (α = .88). Since the reliability coefficient depends on the number of the items the questionnaire consists of, a decrease in the number of items leads to a decrease in the reliability for the short version of the TICS. Concerning the factor structure, the nine-item short version of the TICS shows an excellent fit of the one-factor model. There is evidence for strict invariance across gender and age, which is associated with the possibility of an unbiased comparison of means, correlation coefficients, path coefficients within SEM as well as the possibility of undistorted screening decisions between aforementioned groups, which appears to be explicitly relevant.

Concerning the correlations with the scales of the 57-item long form of the TICS, the nine-item short version of the TICS shows higher associations with the scales of the 57-item long form of the TICS than the 12-item SSCS. This is explicable, since four items of the 12-item SSCS alone measure the subscale Chronic Worrying. The heuristic model with the two components High Demands and Lack of Satisfaction is represented by the new nine-item short version of the TICS.

In the development of the short form we tried to avoid committing typical methodological mistakes associated with short-form development which are also called sins [26]. First, authors of questionnaires choose those items with the highest item-total correlations for a given factor [26, 40, 41, 42]. By choosing the highest loading, a high internal consistency estimate of reliability is preserved, but the content domain is inadvertently narrowed and the validity of the short form is lessened [26, 42]. Using the alphamax algorithm, one item of each one of the nine scales of the TICS was present in the nine-item short version, this methodological sin was also not committed. Another proposed sin is the lack of full content coverage of the construct and lack of validity with fewer items. In order to avoid committing this sin, the short form of the TICS with one item from each one of the nine domains of the systemic-requirement-resource model of health [20] was identified. According to the original authors, the content validity of the long form of the TICS bases on the systemic-requirement-resource model of health [20, 19] and, therefore, also of our short version of the TICS.

Furthermore it should be noted that the use of multiple split-half measures (e.g. Cronbach's alpha) in estimating reliability leads to the statistical phenomenon, that reliability will be lower for scales with a few items relative to scales with many items even when they measure the same construct with the same measurement precision. Future Studies should therefore compare the reliability of both short forms (which vary in test length) using cumulative reliability function as recently discussed by Steinborn and Colleagues [43]. Furthermore, due to the aforementioned restrictions of reliability assessment based on Cronbach's alpha, future studies should compare the test-retest reliability of both short forms with each other as well as with the test long form.

Besides the strength of a large sample size, the wide range of ages, and the representative for the general population as a limitation, the results cannot necessarily be applied to highly stressed samples. In turn, the TICS needs to be applied to different professional groups as well as to clinical samples in order to further replicate or reprobate the factorial structure.

The item selection algorithm based on the optimization of Cronbach’s alpha, has the side effect of simultaneously increasing model fit for a one-factorial solution. The better performance of the new nine-item solution compared to the 12-item version is, thus, partly due to the design. In addition, additional instruments for chronic stress should have been implemented to distinguish convergent and divergent validity.

Future research ought to explore which connections exist with other chronic stress questionnaires (convergent validity) and with external ratings (criterion validity). A design with repeated measurements would allow for the comparison of factor structures across time and the determination of possible cohort effects.

## Conclusion

In sum, a reliable nine-item short version of the TICS could be identified for the assessment of chronic stress in all areas. Since we used the proposed approach for the development of short forms by Smith, McCarthy and Andersons [26] and avoided all the sins of short form developers, in our new short version the broad concept of the systemic-requirement-resource model of health on which the TICS bases is represented [20, 19]. Therefore, our short form offers all the validity of the full-length form and fits into large multivariate studies. In addition, this short version shows a very good factorial structure and correlates high with the long version. Due to the given invariance analyses of gender and age specificities in multivariate studies are possible.

### Ethics approval and consent to participate

The study was approved according to the ethical guidelines of the “German Professional Institutions for Social Research” [Arbeitskreis Deutscher Markt- und Sozialforschungsinstitute, Arbeitsgemeinschaft Sozialwissenschaftlicher Institute, Berufsverband Deutscher Markt- und Sozialforscher] as well as by the ethics committee of the University of Leipzig, Germany. Ethics reference number is 050-13-11032013.

## Supporting information

S1 Table Means, Standard deviation, and inter-correlation of the TICS items. (DOCX)

S1 Data Trier Inventory for Chronic Stress (TICS) 57-items. (SAV)

## Abbreviations

CFA: Confirmatory Factor Analysis
CFI: Comparative Fit Index
MLM: Maximum Likelihood Estimation
RMSEA: Root Mean Square Error of Approximation
SRMR: Standardized Root Mean Square Residual
SSCS: Short Screening Scale for Chronic Stress
TICS: Trier Inventory for Chronic Stress
TLI: Tucker-Lewis-Index
