# Supplementary material for: Norm values and psychometric properties of the short version of the Trier Inventory for Chronic Stress (TICS) in a representative German sample
Source: PLoS One. 2019 Nov 18;14(11):e0222277. doi: 10.1371/journal.pone.0222277 (PMC6860447; doi:10.1371/journal.pone.0222277)
Supplement: S1 Table — (DOCX) [file pone.0222277.s001.docx]

|  | M | SD | tics01 | tics02 | tics03 | tics04 | tics05 | tics06 | tics07 | tics08 | tics09 | tics10 | tics11 | tics12 | tics13 | tics14 | tics15 | tics16 | tics17 | tics18 | tics19 | tics20 | tics21 | tics22 | tics23 | tics24 | tics25 | tics26 | tics27 | tics28 | tics29 | tics30 | tics31 | tics32 | tics33 | tics34 | tics35 | tics36 | tics37 | tics38 | tics39 | tics40 | tics41 | tics42 | tics43 | tics44 | tics45 | tics46 | tics47 | tics48 | tics49 | tics50 | tics51 | tics52 | tics53 | tics54 | tics55 | tics56 | tics57 |
| --- | --- | --- | --- | --- | --- | --- | --- | --- | --- | --- | --- | --- | --- | --- | --- | --- | --- | --- | --- | --- | --- | --- | --- | --- | --- | --- | --- | --- | --- | --- | --- | --- | --- | --- | --- | --- | --- | --- | --- | --- | --- | --- | --- | --- | --- | --- | --- | --- | --- | --- | --- | --- | --- | --- | --- | --- | --- | --- | --- |
| tics01 | 1.34 | 0.99 | 1 | .422 | .329 | .441 | .310 | .329 | .394 | .350 | .299 | .126 | .146 | .312 | .276 | .318 | .266 | .310 | .424 | .311 | .390 | .293 | .187 | .332 | .294 | .285 | .324 | .252 | .412 | .377 | .292 | .324 | .295 | .325 | .285 | .188 | .280 | .279 | .273 | .394 | .380 | .265 | .081 | .151 | .349 | .431 | .271 | .336 | .324 | .251 | .362 | .407 | .173 | .230 | .202 | .399 | .282 | .230 | .385 |
| tics02 | 1.29 | 0.98 |  | 1 | .476 | .374 | .393 | .461 | .301 | .350 | .364 | .310 | .228 | .355 | .380 | .340 | .400 | .399 | .336 | .462 | .380 | .352 | .355 | .272 | .232 | .319 | .368 | .371 | .282 | .344 | .402 | .328 | .500 | .277 | .374 | .294 | .374 | .361 | .339 | .341 | .300 | .307 | .246 | .268 | .306 | .385 | .357 | .504 | .319 | .405 | .291 | .328 | .250 | .343 | .383 | .386 | .334 | .345 | .397 |
| tics03 | 0.89 | 0.87 |  |  | 1 | .486 | .395 | .414 | .258 | .353 | .411 | .296 | .279 | .355 | .356 | .347 | .397 | .409 | .370 | .469 | .398 | .552 | .391 | .282 | .252 | .519 | .449 | .423 | .290 | .356 | .402 | .330 | .428 | .270 | .435 | .358 | .501 | .418 | .401 | .393 | .277 | .357 | .294 | .292 | .276 | .406 | .440 | .415 | .427 | .337 | .239 | .358 | .297 | .396 | .338 | .476 | .505 | .326 | .463 |
| tics04 | 1.12 | 0.99 |  |  |  | 1 | .440 | .376 | .368 | .370 | .296 | .166 | .148 | .310 | .344 | .392 | .345 | .283 | .508 | .402 | .436 | .416 | .229 | .337 | .277 | .346 | .293 | .351 | .499 | .420 | .331 | .312 | .372 | .339 | .368 | .221 | .368 | .277 | .391 | .509 | .346 | .328 | .147 | .161 | .349 | .499 | .382 | .418 | .350 | .299 | .313 | .490 | .180 | .334 | .233 | .487 | .382 | .218 | .451 |
| tics05 | 1.21 | 0.97 |  |  |  |  | 1 | .495 | .250 | .338 | .311 | .271 | .147 | .320 | .514 | .397 | .403 | .299 | .427 | .417 | .352 | .391 | .366 | .289 | .245 | .347 | .327 | .383 | .400 | .378 | .319 | .280 | .420 | .305 | .441 | .258 | .369 | .307 | .501 | .436 | .273 | .363 | .262 | .201 | .289 | .406 | .400 | .462 | .326 | .412 | .268 | .381 | .212 | .371 | .322 | .427 | .389 | .244 | .433 |
| tics06 | 1.23 | 0.93 |  |  |  |  |  | 1 | .353 | .406 | .395 | .271 | .209 | .420 | .431 | .404 | .567 | .408 | .411 | .476 | .444 | .408 | .354 | .316 | .285 | .386 | .375 | .542 | .352 | .395 | .416 | .372 | .443 | .301 | .493 | .333 | .418 | .380 | .417 | .417 | .328 | .389 | .292 | .269 | .338 | .419 | .516 | .433 | .326 | .382 | .276 | .345 | .259 | .498 | .368 | .417 | .404 | .295 | .419 |
| tics07 | 1.38 | 1.17 |  |  |  |  |  |  | 1 | .552 | .263 | .022 | .026 | .323 | .255 | .419 | .291 | .214 | .454 | .255 | .505 | .228 | .113 | .543 | .408 | .207 | .209 | .231 | .451 | .482 | .220 | .370 | .228 | .478 | .264 | .093 | .221 | .189 | .262 | .426 | .561 | .372 | .018 | .025 | .553 | .423 | .273 | .296 | .160 | .226 | .566 | .437 | .018 | .203 | .106 | .322 | .195 | .073 | .356 |
| tics08 | 1.31 | 1.02 |  |  |  |  |  |  |  | 1 | .401 | .142 | .099 | .491 | .333 | .501 | .385 | .295 | .437 | .379 | .432 | .353 | .246 | .544 | .521 | .299 | .268 | .332 | .421 | .429 | .303 | .460 | .304 | .507 | .377 | .223 | .347 | .295 | .333 | .416 | .419 | .505 | .161 | .137 | .524 | .420 | .401 | .351 | .245 | .267 | .457 | .401 | .131 | .304 | .231 | .345 | .304 | .195 | .364 |
| tics09 | 1.23 | 0.97 |  |  |  |  |  |  |  |  | 1 | .335 | .292 | .326 | .304 | .294 | .382 | .517 | .308 | .359 | .327 | .404 | .361 | .287 | .206 | .370 | .446 | .372 | .232 | .333 | .387 | .332 | .335 | .257 | .388 | .396 | .404 | .507 | .321 | .301 | .289 | .329 | .294 | .314 | .257 | .328 | .378 | .316 | .375 | .296 | .247 | .259 | .310 | .344 | .337 | .363 | .411 | .332 | .362 |
| tics10 | 1.23 | 1.05 |  |  |  |  |  |  |  |  |  | 1 | .441 | .262 | .259 | .156 | .270 | .354 | .114 | .342 | .169 | .308 | .547 | .092 | .075 | .279 | .352 | .319 | .057 | .143 | .328 | .181 | .328 | .085 | .303 | .391 | .333 | .359 | .303 | .166 | .101 | .233 | .506 | .416 | .038 | .125 | .295 | .302 | .268 | .340 | 0.02 | .067 | .395 | .304 | .483 | .219 | .308 | .377 | .240 |
| tics11 | 1.28 | 1.16 |  |  |  |  |  |  |  |  |  |  | 1 | .333 | .141 | .098 | .199 | .371 | .098 | .242 | .145 | .310 | .400 | .078 | .077 | .269 | .363 | .262 | .039 | .161 | .433 | .225 | .285 | .065 | .259 | .522 | .320 | .365 | .215 | .136 | .058 | .163 | .409 | .580 | .059 | .150 | .257 | .210 | .278 | .208 | .016 | .070 | .585 | .259 | .383 | .239 | .293 | .500 | .223 |
| tics12 | 1.35 | 1.01 |  |  |  |  |  |  |  |  |  |  |  | 1 | .447 | .442 | .376 | .374 | .356 | .396 | .432 | .364 | .346 | .419 | .426 | .324 | .372 | .383 | .323 | .365 | .381 | .489 | .353 | .401 | .359 | .312 | .372 | .347 | .369 | .382 | .332 | .472 | .273 | .278 | .391 | .361 | .401 | .338 | .286 | .328 | .338 | .317 | .270 | .375 | .336 | .365 | .351 | .326 | .361 |
| tics13 | 1.47 | 0.98 |  |  |  |  |  |  |  |  |  |  |  |  | 1 | .493 | .420 | .311 | .382 | .395 | .403 | .368 | .347 | .366 | .279 | .278 | .332 | .374 | .384 | .359 | .308 | .349 | .387 | .355 | .419 | .222 | .377 | .325 | .488 | .439 | .341 | .380 | .272 | .191 | .334 | .413 | .367 | .410 | .300 | .419 | .267 | .360 | .184 | .348 | .349 | .400 | .354 | .243 | .381 |
| tics14 | 1.18 | 1.05 |  |  |  |  |  |  |  |  |  |  |  |  |  | 1 | .512 | .278 | .463 | .436 | .439 | .375 | .280 | .529 | .468 | .327 | .292 | .364 | .496 | .419 | .330 | .455 | .357 | .556 | .429 | .239 | .383 | .270 | .412 | .469 | .372 | .452 | .186 | .167 | .547 | .466 | .410 | .411 | .290 | .305 | .402 | .414 | .141 | .351 | .238 | .390 | .350 | .181 | .394 |
| tics15 | 1.06 | 0.95 |  |  |  |  |  |  |  |  |  |  |  |  |  |  | 1 | .448 | .402 | .462 | .422 | .420 | .357 | .329 | .263 | .366 | .365 | .568 | .346 | .413 | .405 | .387 | .428 | .318 | .554 | .354 | .406 | .373 | .431 | .410 | .307 | .393 | .293 | .265 | .311 | .400 | .555 | .424 | .292 | .335 | .253 | .328 | .255 | .518 | .343 | .386 | .387 | .319 | .411 |
| tics16 | 1.29 | 1.04 |  |  |  |  |  |  |  |  |  |  |  |  |  |  |  | 1 | .366 | .414 | .351 | .431 | .430 | .257 | .208 | .386 | .555 | .402 | .216 | .350 | .444 | .355 | .374 | .211 | .404 | .422 | .414 | .623 | .347 | .311 | .263 | .321 | .346 | .382 | .244 | .353 | .400 | .352 | .404 | .324 | .222 | .257 | .394 | .367 | .421 | .393 | .413 | .417 | .364 |
| tics17 | 1.21 | 1.05 |  |  |  |  |  |  |  |  |  |  |  |  |  |  |  |  | 1 | .479 | .511 | .375 | .232 | .440 | .399 | .329 | .351 | .342 | .629 | .499 | .334 | .394 | .360 | .426 | .413 | .226 | .346 | .300 | .412 | .586 | .433 | .390 | .095 | .119 | .488 | .606 | .428 | .408 | .330 | .311 | .406 | .581 | .148 | .345 | .211 | .500 | .374 | .201 | .465 |
| tics18 | 1.05 | 0.98 |  |  |  |  |  |  |  |  |  |  |  |  |  |  |  |  |  | 1 | .481 | .538 | .447 | .339 | .307 | .443 | .443 | .431 | .346 | .408 | .399 | .349 | .535 | .297 | .452 | .362 | .453 | .416 | .445 | .435 | .311 | .408 | .321 | .281 | .306 | .441 | .483 | .527 | .403 | .414 | .238 | .381 | .301 | .444 | .397 | .478 | .481 | .331 | .465 |
| tics19 | 1.25 | 1.04 |  |  |  |  |  |  |  |  |  |  |  |  |  |  |  |  |  |  | 1 | .449 | .301 | .465 | .380 | .359 | .391 | .367 | .466 | .621 | .366 | .428 | .403 | .400 | .436 | .235 | .364 | .361 | .424 | .522 | .537 | .408 | .165 | .160 | .463 | .513 | .421 | .421 | .306 | .344 | .449 | .478 | .168 | .352 | .278 | .487 | .366 | .211 | .515 |
| tics20 | 0.99 | 0.94 |  |  |  |  |  |  |  |  |  |  |  |  |  |  |  |  |  |  |  | 1 | .477 | .296 | .245 | .574 | .470 | .444 | .309 | .389 | .400 | .347 | .498 | .298 | .481 | .391 | .539 | .458 | .468 | .425 | .261 | .357 | .323 | .316 | .264 | .445 | .478 | .445 | .462 | .366 | .228 | .346 | .324 | .409 | .346 | .507 | .596 | .336 | .461 |
| tics21 | 1.20 | 1.00 |  |  |  |  |  |  |  |  |  |  |  |  |  |  |  |  |  |  |  |  | 1 | .227 | .196 | .387 | .412 | .369 | .161 | .272 | .401 | .282 | .405 | .190 | .392 | .427 | .419 | .414 | .387 | .304 | .179 | .319 | .543 | .437 | .166 | .249 | .388 | .416 | .317 | .415 | .137 | .187 | .417 | .369 | .547 | .331 | .406 | .416 | .336 |
| tics22 | 1.51 | 1.14 |  |  |  |  |  |  |  |  |  |  |  |  |  |  |  |  |  |  |  |  |  | 1 | .563 | .256 | .244 | .246 | .488 | .470 | .297 | .432 | .300 | .619 | .317 | .149 | .297 | .248 | .330 | .454 | .482 | .442 | .080 | .098 | .639 | .437 | .296 | .363 | .234 | .268 | .525 | .443 | .100 | .230 | .187 | .365 | .262 | .162 | .336 |
| tics23 | 1.58 | 1.00 |  |  |  |  |  |  |  |  |  |  |  |  |  |  |  |  |  |  |  |  |  |  | 1 | .264 | .232 | .231 | .422 | .361 | .274 | .444 | .256 | .556 | .310 | .158 | .264 | .209 | .282 | .391 | .373 | .496 | .122 | .098 | .526 | .369 | .267 | .281 | .234 | .239 | .432 | .384 | .112 | .232 | .189 | .299 | .211 | .135 | .279 |
| tics24 | 0.83 | 0.91 |  |  |  |  |  |  |  |  |  |  |  |  |  |  |  |  |  |  |  |  |  |  |  | 1 | .547 | .460 | .305 | .358 | .405 | .335 | .501 | .270 | .462 | .457 | .580 | .450 | .432 | .395 | .272 | .359 | .379 | .354 | .246 | .413 | .482 | .413 | .502 | .357 | .197 | .312 | .324 | .400 | .348 | .510 | .580 | .299 | .456 |
| tics25 | 1.10 | 1.03 |  |  |  |  |  |  |  |  |  |  |  |  |  |  |  |  |  |  |  |  |  |  |  |  | 1 | .499 | .301 | .367 | .453 | .359 | .444 | .221 | .435 | .442 | .474 | .619 | .410 | .398 | .297 | .348 | .388 | .396 | .259 | .429 | .447 | .396 | .456 | .354 | .233 | .320 | .377 | .390 | .425 | .476 | .486 | .412 | .452 |
| tics26 | 0.99 | 0.97 |  |  |  |  |  |  |  |  |  |  |  |  |  |  |  |  |  |  |  |  |  |  |  |  |  | 1 | .381 | .394 | .432 | .360 | .460 | .266 | .561 | .431 | .472 | .442 | .484 | .424 | .277 | .384 | .362 | .359 | .266 | .396 | .598 | .455 | .355 | .370 | .220 | .288 | .330 | .588 | .384 | .417 | .449 | .348 | .433 |
| tics27 | 1.34 | 1.09 |  |  |  |  |  |  |  |  |  |  |  |  |  |  |  |  |  |  |  |  |  |  |  |  |  |  | 1 | .580 | .323 | .377 | .352 | .506 | .372 | .195 | .335 | .253 | .423 | .587 | .444 | .405 | .088 | .095 | .552 | .624 | .391 | .426 | .294 | .269 | .413 | .596 | .101 | .337 | .186 | .488 | .322 | .153 | .455 |
| tics28 | 1.19 | 1.03 |  |  |  |  |  |  |  |  |  |  |  |  |  |  |  |  |  |  |  |  |  |  |  |  |  |  |  | 1 | .456 | .428 | .408 | .415 | .423 | .278 | .381 | .388 | .465 | .544 | .559 | .420 | .175 | .187 | .494 | .530 | .469 | .450 | .326 | .319 | .436 | .502 | .186 | .372 | .294 | .493 | .377 | .222 | .558 |
| tics29 | 1.22 | 0.98 |  |  |  |  |  |  |  |  |  |  |  |  |  |  |  |  |  |  |  |  |  |  |  |  |  |  |  |  | 1 | .465 | .443 | .259 | .420 | .493 | .439 | .475 | .407 | .406 | .303 | .383 | .369 | .509 | .310 | .400 | .470 | .416 | .385 | .334 | .252 | .331 | .501 | .412 | .450 | .454 | .410 | .476 | .416 |
| tics30 | 1.36 | 0.99 |  |  |  |  |  |  |  |  |  |  |  |  |  |  |  |  |  |  |  |  |  |  |  |  |  |  |  |  |  | 1 | .389 | .425 | .406 | .295 | .352 | .349 | .364 | .423 | .410 | .462 | .225 | .275 | .475 | .418 | .398 | .340 | .276 | .286 | .412 | .354 | .233 | .346 | .299 | .382 | .336 | .301 | .367 |
| tics31 | 1.05 | 0.99 |  |  |  |  |  |  |  |  |  |  |  |  |  |  |  |  |  |  |  |  |  |  |  |  |  |  |  |  |  |  | 1 | .348 | .486 | .380 | .499 | .411 | .470 | .426 | .334 | .376 | .365 | .347 | .297 | .437 | .480 | .597 | .403 | .403 | .244 | .356 | .336 | .431 | .428 | .473 | .477 | .364 | .473 |
| tics32 | 1.41 | 1.15 |  |  |  |  |  |  |  |  |  |  |  |  |  |  |  |  |  |  |  |  |  |  |  |  |  |  |  |  |  |  |  | 1 | .381 | .168 | .306 | .224 | .341 | .464 | .458 | .492 | .103 | .084 | .637 | .443 | .316 | .356 | .237 | .255 | .483 | .440 | .102 | .249 | .187 | .353 | .278 | .144 | .347 |
| tics33 | 1.04 | 0.99 |  |  |  |  |  |  |  |  |  |  |  |  |  |  |  |  |  |  |  |  |  |  |  |  |  |  |  |  |  |  |  |  | 1 | .481 | .492 | .440 | .491 | .478 | .332 | .440 | .355 | .349 | .366 | .470 | .581 | .461 | .374 | .375 | .271 | .385 | .335 | .564 | .418 | .451 | .460 | .367 | .470 |
| tics34 | 0.94 | 1.03 |  |  |  |  |  |  |  |  |  |  |  |  |  |  |  |  |  |  |  |  |  |  |  |  |  |  |  |  |  |  |  |  |  | 1 | .526 | .470 | .369 | .282 | .171 | .336 | .454 | .611 | .180 | .305 | .467 | .323 | .381 | .271 | .098 | .193 | .578 | .430 | .443 | .370 | .432 | .463 | .366 |
| tics35 | 0.95 | 0.96 |  |  |  |  |  |  |  |  |  |  |  |  |  |  |  |  |  |  |  |  |  |  |  |  |  |  |  |  |  |  |  |  |  |  | 1 | .547 | .478 | .413 | .276 | .412 | .408 | .413 | .310 | .441 | .489 | .451 | .491 | .360 | .225 | .340 | .372 | .450 | .413 | .502 | .581 | .376 | .458 |
| tics36 | 1.17 | 1.07 |  |  |  |  |  |  |  |  |  |  |  |  |  |  |  |  |  |  |  |  |  |  |  |  |  |  |  |  |  |  |  |  |  |  |  | 1 | .460 | .362 | .284 | .363 | .402 | .454 | .269 | .386 | .454 | .389 | .457 | .316 | .210 | .286 | .415 | .414 | .461 | .459 | .464 | .430 | .422 |
| tics37 | 1.06 | 0.98 |  |  |  |  |  |  |  |  |  |  |  |  |  |  |  |  |  |  |  |  |  |  |  |  |  |  |  |  |  |  |  |  |  |  |  |  | 1 | .601 | .350 | .423 | .331 | .315 | .354 | .462 | .507 | .484 | .394 | .432 | .278 | .402 | .295 | .452 | .406 | .504 | .477 | .324 | .526 |
| tics38 | 1.24 | 1.03 |  |  |  |  |  |  |  |  |  |  |  |  |  |  |  |  |  |  |  |  |  |  |  |  |  |  |  |  |  |  |  |  |  |  |  |  |  | 1 | .534 | .465 | .186 | .213 | .525 | .625 | .482 | .489 | .391 | .366 | .426 | .600 | .196 | .391 | .295 | .552 | .421 | .253 | .534 |
| tics39 | 1.33 | 1.18 |  |  |  |  |  |  |  |  |  |  |  |  |  |  |  |  |  |  |  |  |  |  |  |  |  |  |  |  |  |  |  |  |  |  |  |  |  |  | 1 | .491 | .091 | .095 | .527 | .471 | .347 | .376 | .250 | .307 | .576 | .469 | .103 | .242 | .217 | .400 | .259 | .171 | .437 |
| tics40 | 1.13 | 1.01 |  |  |  |  |  |  |  |  |  |  |  |  |  |  |  |  |  |  |  |  |  |  |  |  |  |  |  |  |  |  |  |  |  |  |  |  |  |  |  | 1 | .333 | .253 | .478 | .433 | .459 | .419 | .299 | .350 | .403 | .398 | .220 | .368 | .321 | .387 | .368 | .276 | .412 |
| tics41 | 1.09 | 1.04 |  |  |  |  |  |  |  |  |  |  |  |  |  |  |  |  |  |  |  |  |  |  |  |  |  |  |  |  |  |  |  |  |  |  |  |  |  |  |  |  | 1 | .564 | .057 | .164 | .388 | .326 | .307 | .345 | .023 | .090 | .450 | .368 | .515 | .281 | .364 | .418 | .270 |
| tics42 | 1.18 | 1.07 |  |  |  |  |  |  |  |  |  |  |  |  |  |  |  |  |  |  |  |  |  |  |  |  |  |  |  |  |  |  |  |  |  |  |  |  |  |  |  |  |  | 1 | .127 | .211 | .363 | .285 | .340 | .260 | .055 | .115 | .675 | .358 | .469 | .308 | .367 | .559 | .277 |
| tics43 | 1.41 | 1.17 |  |  |  |  |  |  |  |  |  |  |  |  |  |  |  |  |  |  |  |  |  |  |  |  |  |  |  |  |  |  |  |  |  |  |  |  |  |  |  |  |  |  | 1 | .547 | .327 | .383 | .229 | .250 | .567 | .512 | .099 | .264 | .200 | .400 | .268 | .148 | .393 |
| tics44 | 1.18 | 1.02 |  |  |  |  |  |  |  |  |  |  |  |  |  |  |  |  |  |  |  |  |  |  |  |  |  |  |  |  |  |  |  |  |  |  |  |  |  |  |  |  |  |  |  | 1 | .524 | .490 | .408 | .316 | .403 | .609 | .221 | .399 | .287 | .585 | .445 | .256 | .522 |
| tics45 | 0.87 | 0.94 |  |  |  |  |  |  |  |  |  |  |  |  |  |  |  |  |  |  |  |  |  |  |  |  |  |  |  |  |  |  |  |  |  |  |  |  |  |  |  |  |  |  |  |  | 1 | .513 | .384 | .340 | .246 | .402 | .365 | .625 | .387 | .484 | .502 | .373 | .514 |
| tics46 | 1.12 | 1.06 |  |  |  |  |  |  |  |  |  |  |  |  |  |  |  |  |  |  |  |  |  |  |  |  |  |  |  |  |  |  |  |  |  |  |  |  |  |  |  |  |  |  |  |  |  | 1 | .379 | .465 | .324 | .433 | .276 | .427 | .405 | .501 | .460 | .349 | .497 |
| tics47 | 1.04 | 0.87 |  |  |  |  |  |  |  |  |  |  |  |  |  |  |  |  |  |  |  |  |  |  |  |  |  |  |  |  |  |  |  |  |  |  |  |  |  |  |  |  |  |  |  |  |  |  | 1 | .388 | .224 | .378 | .380 | .398 | .408 | .537 | .526 | .326 | .447 |
| tics48 | 1.12 | 0.97 |  |  |  |  |  |  |  |  |  |  |  |  |  |  |  |  |  |  |  |  |  |  |  |  |  |  |  |  |  |  |  |  |  |  |  |  |  |  |  |  |  |  |  |  |  |  |  | 1 | .355 | .332 | .290 | .354 | .475 | .421 | .415 | .324 | .401 |
| tics49 | 1.32 | 1.14 |  |  |  |  |  |  |  |  |  |  |  |  |  |  |  |  |  |  |  |  |  |  |  |  |  |  |  |  |  |  |  |  |  |  |  |  |  |  |  |  |  |  |  |  |  |  |  |  | 1 | .509 | .100 | .210 | .182 | .361 | .255 | .175 | .393 |
| tics50 | 1.28 | 1.03 |  |  |  |  |  |  |  |  |  |  |  |  |  |  |  |  |  |  |  |  |  |  |  |  |  |  |  |  |  |  |  |  |  |  |  |  |  |  |  |  |  |  |  |  |  |  |  |  |  | 1 | .211 | .338 | .221 | .552 | .384 | .212 | .501 |
| tics51 | 1.19 | 1.06 |  |  |  |  |  |  |  |  |  |  |  |  |  |  |  |  |  |  |  |  |  |  |  |  |  |  |  |  |  |  |  |  |  |  |  |  |  |  |  |  |  |  |  |  |  |  |  |  |  |  | 1 | .422 | .512 | .329 | .394 | .607 | .306 |
| tics52 | 0.89 | 0.93 |  |  |  |  |  |  |  |  |  |  |  |  |  |  |  |  |  |  |  |  |  |  |  |  |  |  |  |  |  |  |  |  |  |  |  |  |  |  |  |  |  |  |  |  |  |  |  |  |  |  |  | 1 | .477 | .458 | .440 | .385 | .475 |
| tics53 | 1.29 | 1.03 |  |  |  |  |  |  |  |  |  |  |  |  |  |  |  |  |  |  |  |  |  |  |  |  |  |  |  |  |  |  |  |  |  |  |  |  |  |  |  |  |  |  |  |  |  |  |  |  |  |  |  |  | 1 | .449 | .401 | .489 | .384 |
| tics54 | 1.07 | 0.95 |  |  |  |  |  |  |  |  |  |  |  |  |  |  |  |  |  |  |  |  |  |  |  |  |  |  |  |  |  |  |  |  |  |  |  |  |  |  |  |  |  |  |  |  |  |  |  |  |  |  |  |  |  | 1 | .601 | .360 | .589 |
| tics55 | 0.89 | 0.91 |  |  |  |  |  |  |  |  |  |  |  |  |  |  |  |  |  |  |  |  |  |  |  |  |  |  |  |  |  |  |  |  |  |  |  |  |  |  |  |  |  |  |  |  |  |  |  |  |  |  |  |  |  |  | 1 | .425 | .514 |
| tics56 | 1.30 | 1.07 |  |  |  |  |  |  |  |  |  |  |  |  |  |  |  |  |  |  |  |  |  |  |  |  |  |  |  |  |  |  |  |  |  |  |  |  |  |  |  |  |  |  |  |  |  |  |  |  |  |  |  |  |  |  |  | 1 | .397 |
| tics57 | 0.96 | 0.92 |  |  |  |  |  |  |  |  |  |  |  |  |  |  |  |  |  |  |  |  |  |  |  |  |  |  |  |  |  |  |  |  |  |  |  |  |  |  |  |  |  |  |  |  |  |  |  |  |  |  |  |  |  |  |  |  | 1 |
